# Transmission routes maintaining a viral pathogen of steelhead trout within a complex multi‐host assemblage

**DOI:** 10.1002/ece3.3276

**Published:** 2017-09-06

**Authors:** Rachel Breyta, Ilana Brito, Paige Ferguson, Gael Kurath, Kerry A. Naish, Maureen K. Purcell, Andrew R. Wargo, Shannon LaDeau

**Affiliations:** ^1^ Microbiology Oregon State University Corvallis OR USA; ^2^ Cary Institute for Ecosystems Studies Millbrook NY USA; ^3^ Biomedical Engineering Cornell University Ithaca NY USA; ^4^ Biological Sciences University of Alabama Tuscaloosa AL USA; ^5^ US Geological Survey, Western Fisheries Research Center Seattle WA USA; ^6^ School of Aquatic and Fisheries Sciences University of Washington Seattle WA USA; ^7^ Department of Aquatic Health Sciences Virginia Institute of Marine Science Gloucester Point VA USA

**Keywords:** aquatic ecosystem, disease ecology, freshwater ecosystem, host assemblage, infectious hematopoietic necrosis virus, resource management, salmonid fish, steelhead trout, transmission routes

## Abstract

This is the first comprehensive region wide, spatially explicit epidemiologic analysis of surveillance data of the aquatic viral pathogen infectious hematopoietic necrosis virus (IHNV) infecting native salmonid fish. The pathogen has been documented in the freshwater ecosystem of the Pacific Northwest of North America since the 1950s, and the current report describes the disease ecology of IHNV during 2000–2012. Prevalence of IHNV infection in monitored salmonid host cohorts ranged from 8% to 30%, with the highest levels observed in juvenile steelhead trout. The spatial distribution of all IHNV‐infected cohorts was concentrated in two sub‐regions of the study area, where historic burden of the viral disease has been high. During the study period, prevalence levels fluctuated with a temporal peak in 2002. Virologic and genetic surveillance data were analyzed for evidence of three separate but not mutually exclusive transmission routes hypothesized to be maintaining IHNV in the freshwater ecosystem. Transmission between year classes of juvenile fish at individual sites (route 1) was supported at varying levels of certainty in 10%–55% of candidate cases, transmission between neighboring juvenile cohorts (route 2) was supported in 31%–78% of candidate cases, and transmission from adult fish returning to the same site as an infected juvenile cohort was supported in 26%–74% of candidate cases. The results of this study indicate that multiple specific transmission routes are acting to maintain IHNV in juvenile fish, providing concrete evidence that can be used to improve resource management. Furthermore, these results demonstrate that more sophisticated analysis of available spatio‐temporal and genetic data is likely to yield greater insight in future studies.

## 1 INTRODUCTION

Steelhead trout (*Oncorhynchus mykiss*) are a culturally and ecologically important salmonid fish in the Pacific Northwest. Steelhead trout are anadromous and spend much of their life in the ocean although they rely on freshwater habitat during spawning and the initial year of juvenile development. The Columbia River Basin and coastal Washington and Oregon make up a significant portion of the steelhead endemic range in North America, which also includes parts of British Columbia, Alaska, and California (Bootland & Leong, 1999). Several populations of steelhead trout are on the US Endangered Species Act list of threatened species across this region (Gustafson et al., 2007), where a range of strategies including captive rearing efforts are used to try to rebuild particular stocks (Fraser, 2008). In addition to habitat loss due to changes in land‐use and river conditions, including dams, the pathogen infectious hematopoietic necrosis virus (IHNV) is a current and serious threat for steelhead trout (Bootland & Leong, 1999; Breyta, Jones, & Kurath, 2014; Breyta et al., 2013; Williams & Amend, 1976; Wolf, 1988). This virus was historically observed to cause disease predominantly in sockeye salmon (*Oncorhynchus nerka*) (Meyers, Thomas, Follett, & Saft, 1990; Williams & Amend, 1976), but it emerged by a host jump into farmed rainbow trout (freshwater resident *O. mykiss*) in the 1970s (Amend, 1975; Kurath et al., 2003; Troyer, LaPatra, & Kurath, 2000) and spread through Columbia River Basin steelhead populations since the 1980s (Breyta, Black, Kaufman, & Kurath, 2016; Groberg, Hedrick, & Fryer, 1982). Also in the 1980s, IHNV in the Columbia River Basin adapted to increase prevalence in Chinook salmon (*Oncorhynchus tshawytscha*) (Arkush, Mendonca, McBride, & Hedrick, 2004; Black, Breyta, Bedford, & Kurath, 2016), which are often reared with steelhead trout and share similar spawning run timing. This is a complex landscape and interactions between disease, habitat changes, and human actions all likely influence steelhead population dynamics. In this paper, we describe the prevalence of IHNV in steelhead and other sympatric Pacific salmonids (*Oncorhynchus* spp.), across the Columbia River Basin and adjacent coastal rivers during the period from 2000 to 2012, and evaluate a suite of predictor variables for explaining juvenile infection rates and epidemiologic patterns across the landscape.

Landscape ecology of infectious disease is an active field of research requiring detailed knowledge of temporal and spatial patterns of pathogen occurrence, and scientifically sound understanding of host–pathogen interactions. The pathogen in this landscape ecology study, IHNV, causes both acute lethal disease associated with necrosis of the hematopoietic kidney and spleen tissues in juvenile fish, and asymptomatic infection in adult Pacific salmon and trout (*Oncorhynchus* spp.) (Bootland & Leong, 1999; Wolf, 1988). Viral infection is observed in both cultured fish in hatcheries and fish farms as well as wild fish. The virus can be transmitted horizontally via waterborne virus shed by infected fish and from parent to offspring by egg‐ or sperm‐associated viral exposure. However, in cultured fish populations, transmission from parent to offspring is effectively eliminated by the standard practice of disinfecting fertilized eggs with iodophor (Meyers et al., 1990). Cultured fish, therefore, are most at risk of IHNV transmission when infected fish shed active virus into the water supply, or when challenges arise in implementing biosecurity protocols.

This study is focused on IHNV epidemiology within the region consisting of the coastal watersheds of Oregon and Washington (excluding Puget Sound), and the Columbia River Basin, which represents a large watershed draining an area of 668,000 km^2^ including most of inland Washington, Oregon, and Idaho. Within this study region, there have been several recorded high‐impact IHNV emergence events (Bootland & Leong, 1999; Breyta et al., 2013; Breyta, Black, et al., 2016; Groberg et al., 1982). There are extensive state, federal, and tribal hatchery culture programs that rear fish at freshwater sites in support of conservation goals or mitigation of habitat loss through activities such as hydroelectric power generation (Naish et al., 2007). Hatchery fish are released as juveniles to migrate to the Pacific Ocean as part of their natural anadromous life cycles. Fish of various species co‐mingle during freshwater migration and in the marine environment for 2–4 years before their return migrations to spawn as mature adults in their natal hatcheries. Wild fish are sympatric with hatchery fish throughout much of their life cycles and transmission of viruses between wild and cultured fish has been documented (Anderson, Engelking, Emmenegger, & Kurath, 2000; Kurath & Winton, 2011). Since hatchery fish are neither wild nor fully domesticated (like farm fish), we use the term “semi‐cultured” to describe the fact that they spend part of their life history in cultured environments, and part in natural environments. Numerous salmonid species that co‐occur in the study region can be infected with IHNV with varying efficiencies (Bootland & Leong, 1999). We focus on the two species that are the most abundant IHNV‐susceptible hosts in the study region, *O. mykiss* and *O. tshawytscha*. The species *O. mykiss* occurs as two distinct life history variants: steelhead and rainbow trout (anadromous and freshwater resident forms, respectively). The species *O. tshawytscha* occurs as life history variants commonly referred to as spring and fall Chinook salmon. For simplicity, these two main host species will be referred to hereafter as steelhead and Chinook, except where rainbow trout is specifically noted.

The genetic diversity of IHNV viruses isolated from fish within the study region also varies spatially and temporally, as indicated by an established genotyping system based on genetic sequences of the variable 303 nt “midG” region in the viral glycoprotein gene (Kurath et al., 2003). A genetic surveillance program for monitoring of IHNV virus genotypes in North America has been conducted at the US Geological Survey, Western Fisheries Research Center (USGS WFRC), including data from virus isolates collected from 1958 to 2016. Over 3,000 virus isolates from fish sampled in Alaska, Washington, Oregon, Idaho, California, and Montana have been analyzed, and the data are publicly available as the MEAP‐IHNV database (Molecular Epidemiology of Aquatic Pathogens‐IHNV; http://gis.nacse.org/ihnv/). This typing program has detected 322 unique IHNV genotypes to date (Breyta, Black, et al., 2016; Kurath et al., 2003), falling into three major IHNV genogroups in North America, U, M, and L. Within our study region, both U and M group viruses co‐occur (Breyta, Black, et al., 2016; Garver, Troyer, & Kurath, 2003). Although virus isolates from each genogroup have been demonstrated to infect all salmonid host species tested to date, they differ in host‐specific fitness and virulence. For example, U virus are most fit and virulent in sockeye, whereas M viruses are most fit and virulent in steelhead and rainbow trout (Breyta et al., 2014; Garver, Batts, & Kurath, 2006; Peñaranda, Purcell, & Kurath, 2009; Peñaranda, Wargo, & Kurath, 2011; Purcell, Garver, Conway, Elliott, & Kurath, 2009). Phenotypic variation in host specificity of U and M virus types is an essential aspect of the complex ecology of IHNV in the study region. The majority of disease impacts in the study area are due to M genotype viruses in steelhead trout (Breyta, Black, et al., 2016). In coastal watersheds of Oregon and Washington, IHN disease has historically been all due to U genogroup viruses in sockeye salmon (*O. nerka*) (Emmenegger & Kurath, 2002) although disease outbreaks due to M genogroup types were detected during a major IHNV emergence in coastal steelhead during 2007–2011 (Breyta et al., 2013).

In addition to the MEAP‐IHNV genotyping database just described, a novel database of IHNV virological surveillance data (IHNV‐VGS database) has recently been created (Breyta, Brito, Kurath, & LaDeau, 2017), including both positive and negative virus testing results for the years 2000–2012. The IHNV‐VGS database was used here to (1) analyze steelhead and sympatric salmonids testing effort and infection rates, (2) evaluate spatial and temporal patterns of IHNV prevalence, and (3) evaluate support for three hypothesized transmission pathways that may be responsible for IHNV infection in juvenile hatchery fish.

## 2 METHODS

Surveillance and genotyping records from the Columbia River Basin and Washington and Oregon coastal region were obtained from the IHNV‐VGS surveillance and genotyping database (Breyta et al., 2017). Briefly, the IHNV‐VGS database records for fish sampled at hatchery‐related sites include life stage, indicated as juvenile or returning adult. Surveillance testing utilizes the validated two‐stage virus detection method with high sensitivity and specificity (Purcell et al., 2013) and is most frequently deployed in a manner that reliably detects prevalence at or above 5%. While viral loads in individual fish are not dependent on life stage, population‐level prevalence levels likely fluctuate due to a number of factors. As a result, within‐population prevalence levels were not used as a variable in these studies. Fish cohorts are defined by year, age class, species, run timing, and location. The database also includes data on wild fish sampled by the National Wild Fish Health Survey (NWFHS), available at https://www.fws.gov/wildfishsurvey, but these records do not have age‐specific information. Each database record was assigned to one of the 123 sub‐regional watersheds (USGS 8‐digit hydrological unit codes, HUC8, National Hydrography Dataset https://nhd.usgs.gov/, in hydrological region 17, hereafter designated “HUC8 watersheds”). Most hatcheries routinely test adult fish at spawning for a suite of pathogens, including IHNV. The majority of juvenile fish testing is conducted in cases of symptomatic disease, and so the absence of reported juvenile testing at any site is assumed to reflect an absence of IHN disease (assumption validated via personal communication with J. Thomas, WDFW; M. Blair, USFWS; B. Stewart, NWIFC; and D. Munson, IDFG). Because adults infected with IHNV are usually asymptomatic, missing adult testing data provide no such inference regarding IHNV status.

The USGS WFRC conducts IHNV genotyping by midG (303 nt) sequence analysis of IHNV field isolates for fisheries managers throughout the Pacific Northwest. The data are maintained for stakeholders online, and methods have been described previously (Emmenegger & Kurath, 2002; Emmenegger, Meyers, Burton, & Kurath, 2000; Kurath et al., 2003). Briefly, each genetic variant is a specific individual sequence (e.g., differs by at least 1nt from all known midG sequences), hereafter referred to as a genotype. In the IHNV‐VGS database, genotype data were available for virus isolates from 66% of all positive fish cohorts detected by hatchery site sampling (no virus isolates from wild fish were submitted for genotyping). Some satellite sampling locations (see definition below) were reported with ambiguous locations; therefore, these sites were assigned a geospatial location 1 km upstream from the most downstream junction associated with the written description of the site.

We analyzed the database for evidence that contact between specific age classes or recurrence at particular sites was associated with IHNV infection in hatchery‐reared juvenile fish. Although we included juvenile fish of any species, the majority of positive juvenile cohorts were steelhead (Table 1). Below we evaluate relative support for three general and not mutually exclusive transmission scenarios: (1) between juvenile cohorts reared in the same hatchery (consecutive or concurrent cohorts at same location), (2) between juvenile cohorts at nearby hatcheries (same year but different locations within HUC8 watersheds), and (3) from adult fish returning to a hatchery site (same or previous year). Initial analyses were performed with prevalence data, after which genotyping data were queried for additional support. Much of the epidemiologic inference drawn from genotyping data depends on the relative uniqueness of a given genotype. Thus, relatively rare genotypes can provide strong indications of transmission links, whereas common or dominant genotypes are less informative because they may be observed in several possible transmission source populations. However, even the dominant types described here can be informative if spatial and temporal data are considered, for instance if a dominant genotype is found outside its previously observed spatial or temporal distribution. In addition, a finding of unmatched genotypes is a contra‐indication of a specific transmission link regardless of whether the genotypes are common or rare.

**Table 1:** Infectious hematopoietic necrosis virus (IHNV) testing effort and prevalence by host type and age class for hatchery sites

Host type^a^	Adult fish			Juvenile fish		
	Records	IHNV+ records	Prevalence (by records) (%)	Records	IHNV+ records	Prevalence (by records) (%)
Steelhead trout	820	252	30.7	373	95	25.5
Rainbow trout	240	25	10.4	254	37	14.6
Chinook salmon	1,027	271	26.4	544	45	8.3
Sockeye salmon	68	22	32.4	64	7	10.9
Kokanee salmon	151	15	9.9	17	2	11.8
Coho salmon	445	23	5.2	165	1	0.6
Non‐focal hosts^b^	330	17	5.2	137	3	2.2

a Species for host types: steelhead and rainbow trout (anadromous and freshwater forms of *Oncorhynchus mykiss*); Chinook salmon (*Oncorhynchus tshawytscha*); sockeye and kokanee salmon (anadromous and freshwater forms of *Oncorhynchus nerka*); coho salmon (*Oncorhynchus kisutch*).

b Non‐focal hosts include seven species: cutthroat trout (*Oncorhynchus clarkii*), pink salmon (*Oncorhynchus gorbuscha*), chum salmon (*Oncorhynchus keta*), bull trout (*Salvelinus confluentus*), brook trout (*Salvelinus fontinalis*), brown trout (*Salmo trutta*), and Atlantic salmon (*Salmo salar*).

To assess support for pathway 1, we first quantified the recurrence of juvenile infections within a hatchery across years. Furthermore, we assessed hatchery characteristics that were associated with high hatchery infection rates, on the hypothesis that larger hatchery programs would be more likely to support route 1 transmission. We specifically examined how hatchery program size, including numbers of fish and species reared, was associated with the prevalence of juvenile infections within a hatchery across years. We identified clusters of infection across sites within a HUC8 watershed to evaluate support for pathway 2. Finally, the potential role for adult transmission to juvenile cohorts (route 3) was informed by the frequency of consecutive adult and juvenile infections at a particular site and the identity of viral genotypes across host life stages.

In order to evaluate whether or not hatchery program size influences the frequency of juvenile cohort infections, we obtained the numbers of juvenile fish released from hatcheries each year from the Regional Mark Processing Center (RMPC, www.rmpc.org) database. These data were available for 86 of the 132 hatcheries in our dataset that reported testing steelhead and/or Chinook. Juvenile fish release numbers were not available for all 13 years of the study across the 86 hatcheries; thus, a site average was calculated to represent mean hatchery production size during the study period. The average size of a juvenile steelhead cohort was 212,000 (range: 3,000–1,347,000), and the average number of juvenile Chinook released as a cohort was 1,343,000 (range: 26,000–12,067,000). For each hatchery site, we calculated the total number of years, out of 13, that a cohort was recorded as IHNV positive. Unless otherwise noted, this frequency of infected juvenile cohorts for each site was considered the response variable of interest. Maps were created using the WGS1984 projection in ArcGIS. Statistical analyses were performed using the statistical program R (R Core Team, 2014) and included linear (lm) and generalized linear (glm, with Poisson link) regression models.

## 3 RESULTS

### 3.1 Sampling effort

The 6,766 IHNV testing records in the IHNV‐VGS database were from fish sampled at 1,142 unique sites. These included 169 hatchery‐based sites (referred to hereafter as “hatchery sites”), which included 121 hatchery facilities and 48 hatchery satellite locations such as fish traps, weirs, or fish ladders. The records from hatchery‐based sites were predominantly from testing of hatchery‐origin fish and comprise the majority of the data (3,896 records, or 58%). The greatest proportion of these hatchery origin records were from sites in Washington State (52%), with 31% and 17% from Oregon and Idaho, respectively. Other sites (9% of records) included 12 privately owned fish farms and 144 unstructured sites such as creeks or lakes where fish (of various hatchery, natural, or unknown origin) were sampled as part of general fish health management. Records from fish known to be wild were from 820 sites sampled by the National Wild Fish Health Survey (NWFHS, 2,077 records, with no age‐related information, 33% of the database). Records from testing of steelhead trout comprise 22% of the database (1,485 records, 1,220 with age class specified for fish from hatchery‐related sites). Surveillance effort for the entire database, indicated as the average number of records per year, was 356 (±50 *SD*) for hatchery, farm, or unstructured sites, and 160 (±66 *SD*) for wild fish sites sampled by the NWFHS.

Adult fish testing at hatchery sites was reported in an average 10.3 (±4.1 *SD*) years per site during the 13‐year focal study period. Among the 169 hatchery sites, 62 (37%) reported IHNV‐positive adult cohorts in at least 1 year and 15 (9%) reported positive cohorts in ≥10 years of the study. The average number of years with juvenile testing data was 6.3 (±4.3 *SD*) per site. A majority of hatcheries (111; 65.7%) reported juvenile testing in at least 1 year and 13 hatcheries (8%) reported testing juveniles in all 13 years. Among the 169 hatchery sites, 53 (31%) reported IHNV‐positive juvenile cohorts in at least 1 year and one (0.5%) reported positive cohorts in ≥10 years of the study. Approximately, one‐third of hatcheries (58; 34%) never reported juvenile testing, indicating absence of suspected juvenile infection at those sites. At hatchery sites steelhead adult testing occurred in an average of 8.1 (±4.5) years, and testing of juvenile steelhead occurred in an average 4.5 (±3.9) years across hatchery sites. Adult hatchery‐reared Chinook were tested in an average of 8.8 (±4.8) years, and juvenile fish in an average of 5.1 (±4.3) years across hatchery sites. Wild fish were sampled across 79 of the 121 HUC8 watersheds in the focal region; hatchery‐based records came from 53 HUC8 watersheds. The number of hatcheries per HUC8 watershed ranged from 1 to 4, with a mean of 1.8 (±1.0 *SD*).

Viral genotypes were available for 423 (68%) of the positive adult records and 134 (71%) of the positive juvenile records, accounting for 66% of the 842 positive cohort records in the database. Isolates from steelhead (45%) and Chinook (38%) constituted the majority of genotyped records, with the remaining 17% from other salmonid species. Isolates were genotyped from 87 (52%) of the hatchery sites at least once during the study period. No genotype data were available for the 27 virus‐positive records from the NWFHS wild fish testing.

### 3.2 Virus prevalence in different geographic regions and host types

**Figure 1:**
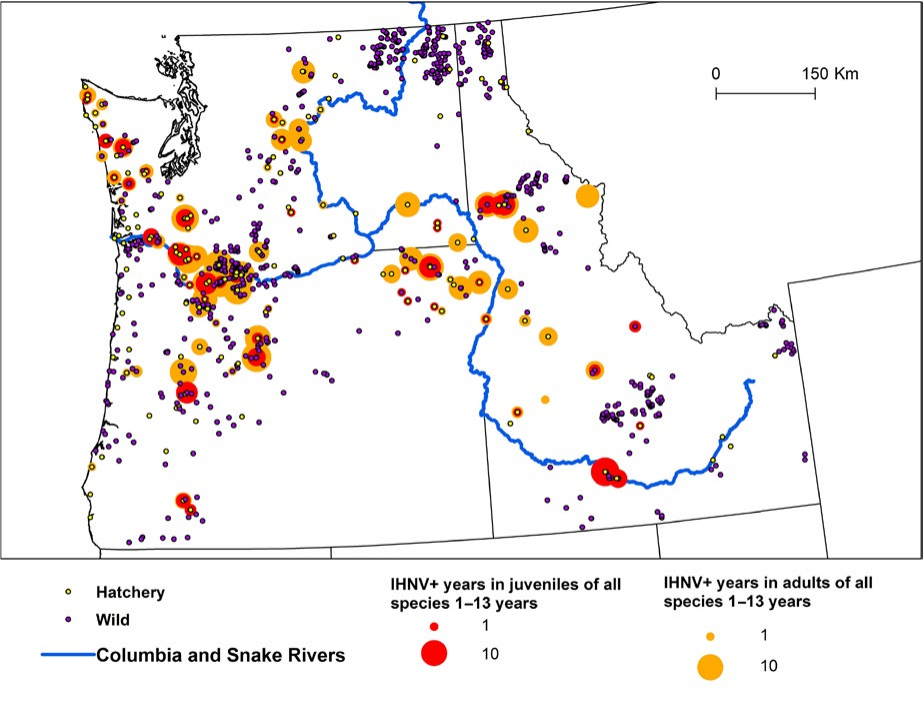
Overall infectious hematopoietic necrosis virus (IHNV) endemicity in the Pacific Northwest. A map of the Pacific Northwest depicting IHNV prevalence over the 2000–2012 time period per hatchery site (yellow circles) and wild site (purple circles). Sites where virus was detected are surrounded by rings scaled by number of years with IHNV detected in adults (orange) and juveniles (red). Also depicted are the Columbia and Snake Rivers (blue)

The overall prevalence for the entire database was 846 IHNV‐positive records out of a total of 6,766 unique testing records (13%). Prevalence was widespread across the region although spatial heterogeneity is evident (Figure 1). Detected prevalence of IHNV during our study period was concentrated most heavily in the lower Columbia and lower Snake River sub‐regions, and was lowest in Oregon coastal watersheds. Summary statistics for adult and juvenile age classes of the six host types with the highest prevalence and testing rates are shown in Table 1. Steelhead and Chinook were the most frequently sampled overall, whereas IHNV prevalence was highest in adult steelhead, Chinook, and sockeye salmon (ranging from 26% to 32%; Table 1). Infection prevalence in steelhead juveniles (26%) was higher than for any other juvenile host type.

**Figure 2:**
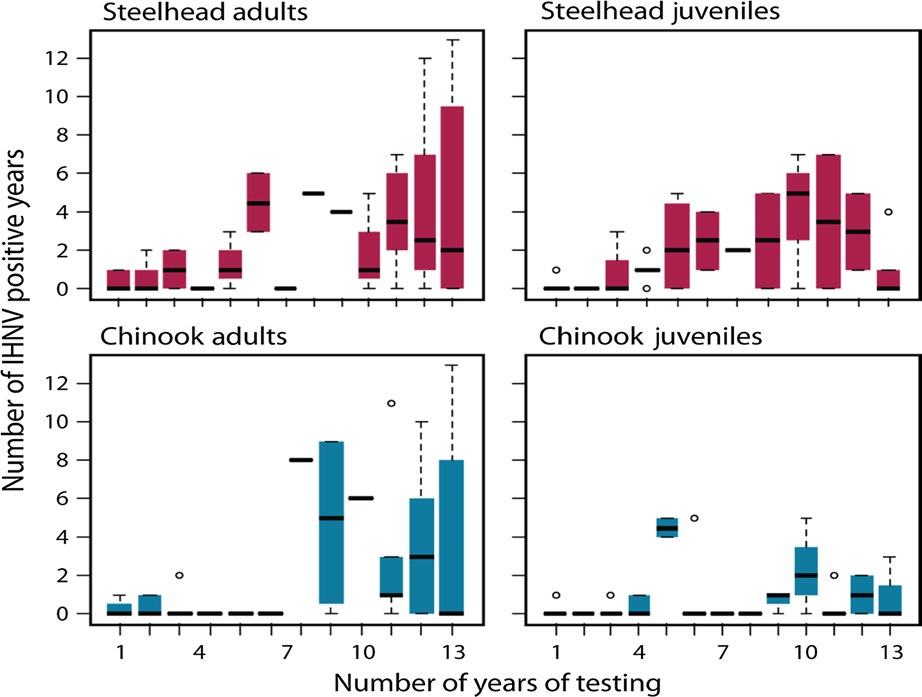
The number of positive records per hatchery site (*y*‐axis) is compared to the total numbers of years each site reported testing (*x*‐axis). Boxplots show the range of positive cohort data for each category of testing frequency. Median values are shown as solid black lines, outliers as open circles

The data imply that adult sampling, while extensive, is not saturated in terms of detecting prevalence. Hatcheries where testing of adult IHNV infection in more years were more likely to report positive adults, suggesting that additional adult sampling could result in greater prevalence (Figure 2). Increasing positive tests for IHNV among juvenile steelhead or Chinook does not increase similarly with testing effort (Figure 2), which is consistent with our assumption that infected juveniles are symptomatic and testing is likely to occur if IHNV is present.

**Figure 3:**
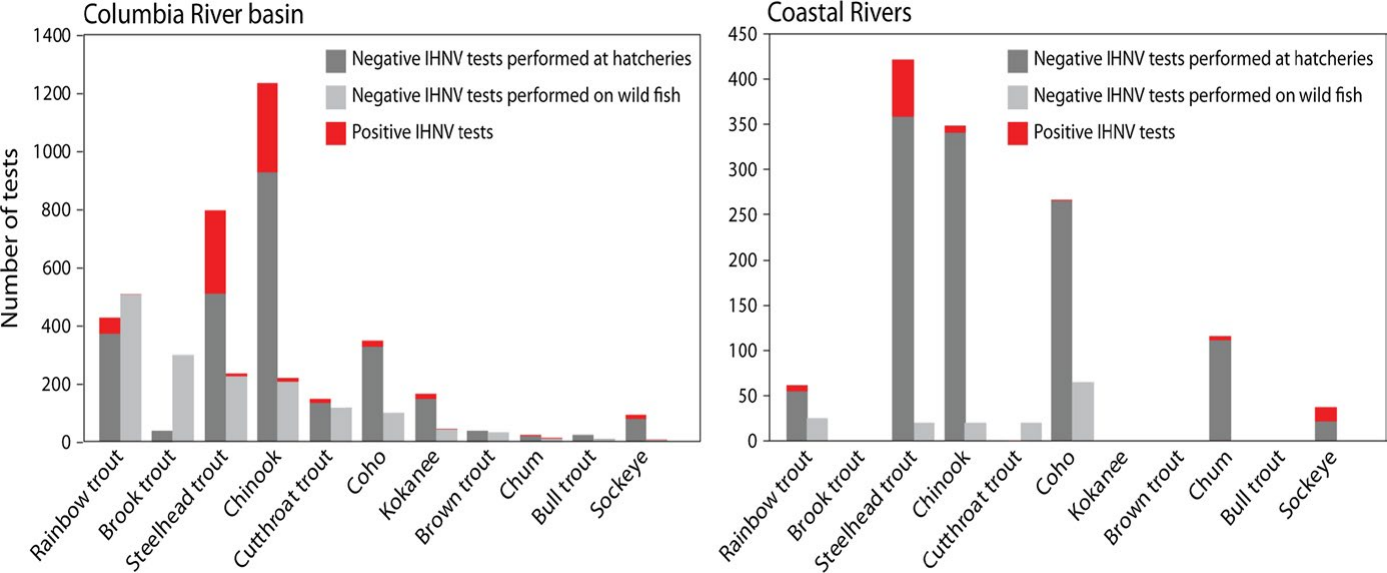
Distribution of testing effort and virus prevalence among different host fish. The different missions across hatchery programs and the National Wild Fish Health Survey result in variation in which species are tested for infectious hematopoietic necrosis virus (IHNV) and how often. Differences in testing profiles are also evident between regions, as shown here for the Columbia River Basin and Coastal Rivers of Washington and Oregon

Overall prevalence of IHNV in any host was significantly higher from hatchery‐based (18%) versus wild fish (1%) samples (χ^2^ < .001). However, this difference is likely to be due in part to differences in sampling effort across wild‐ and hatchery‐reared species. Rainbow trout and coho salmon were the predominant hosts sampled in wild testing efforts for the CRB and coastal river sites, respectively (Figure 3). In contrast, hatchery‐based sampling was significantly weighted toward Chinook and steelhead, which also had the highest prevalence of virus in both the CRB and coastal sites. Although Chinook and steelhead were less frequently sampled in wild fish testing relative to other potential host species, 21 out of 27 virus‐positive records from wild fish occurred in these two species.

### 3.3 Virus prevalence over time

**Figure 4:**
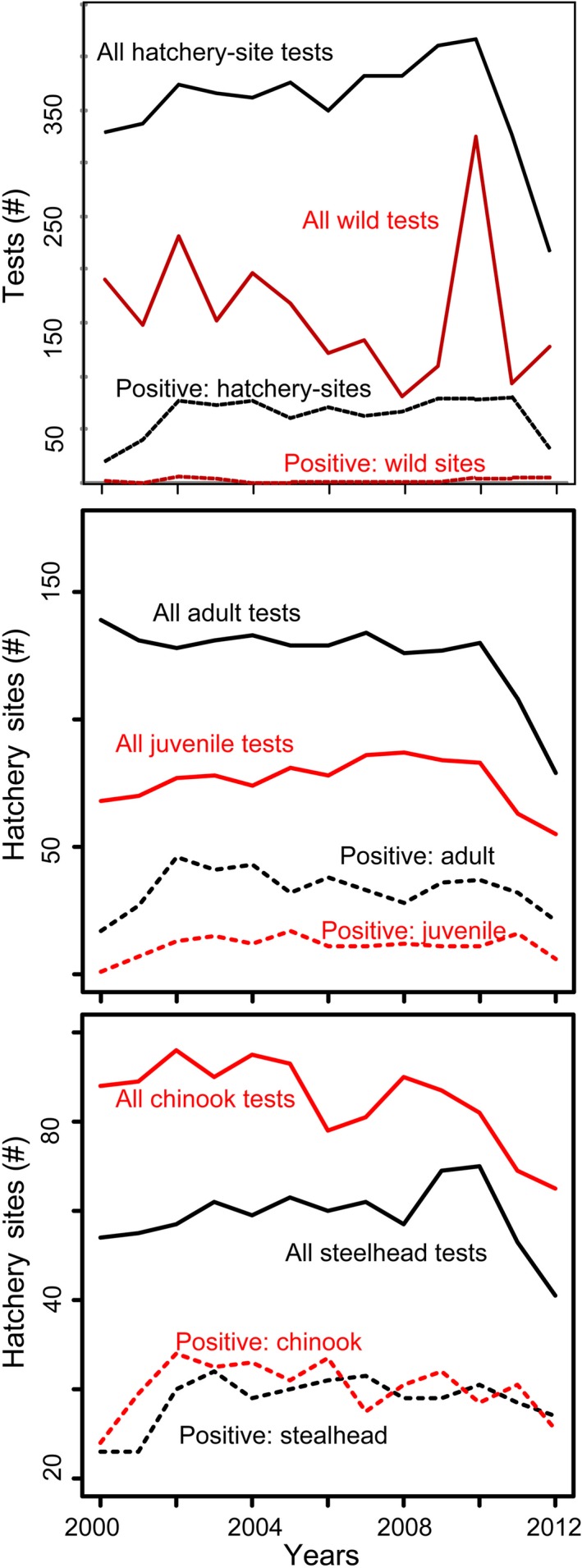
Surveillance and prevalence of infectious hematopoietic necrosis virus (IHNV) over time from 2000 to 2012, from sampling of adult (black) and juvenile (red) fish. The number of cohort‐sites tested (solid lines) relative to the number of IHNV‐positive surveillance tests (dashed lines) during each year of the study period. The top panel depicts these values for tests performed at hatchery or wild sites, the middle panel shows these values for adult or juvenile fish, and the bottom panel compares tests performed on Chinook (*Oncorhynchus tshawytscha*), versus steelhead and rainbow trout combined (both *Oncorhynchus mykiss*)

Prevalence of IHNV in juvenile steelhead was greater than 30% across hatchery sites for the majority of the study period, especially after 2002 (Figure 4). The dramatic rise from 0% to a peak at 35% in 2002 in juvenile steelhead coincided with the emergence of a specific genotype within the M genogroup, which was shown to be more virulent in steelhead relative to earlier viral forms (Breyta, McKenney, Tesfaye, Ono, & Kurath, 2016; Breyta, Samson, Blair, Black, & Kurath, 2016). Juvenile Chinook prevalence has been both lower and more variable, ranging between 0 and a peak at 16% during the study period. Prevalence across adult fish sampled at hatcheries has been more consistent, hovering around 40% for steelhead and just under 30% for Chinook. There is a visible decline in testing records in 2012 across age and species, possibly due to incomplete reporting, fewer fish returning, or fewer tests conducted. It is unclear whether declines in prevalence are due to one or more of these possibilities. Figure 4 highlights the extensive genotyping coverage of positive samples through time. On average, viral genotypes were available for 57% (±25%) of positive adult and 66% (±30%) of juvenile Chinook samples and 75% (±12%) and 78.8% (21%) of positive adult and juvenile steelhead samples. Samples from 100% of positive site‐cohorts were genotyped in 2 years for Chinook juveniles and 3 years for steelhead juveniles (red bars).

### 3.4 Viral genotype diversity

**Figure 5:**
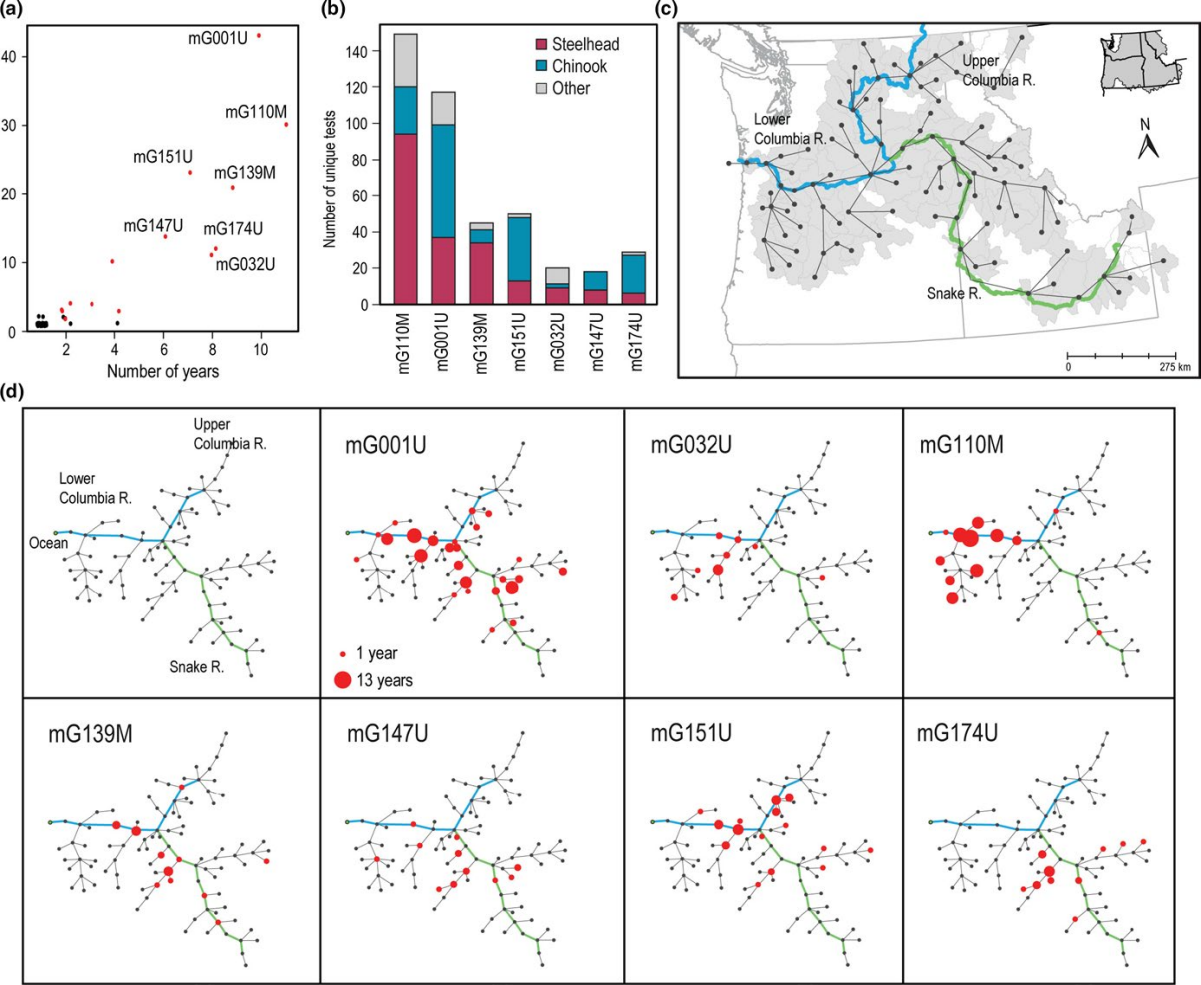
Infectious hematopoietic necrosis virus (IHNV)‐dominant genotypes observed within a river connectivity network across the Columbia River Basin. (a) Spatial and temporal frequency of detection of IHNV genotypes, [no. of sites on *y*‐axis and no. of years on *x*‐axis] revealing a natural breakpoint (≥10 sites and ≥5 years) separating seven dominant from non‐dominant genotypes (only dominant types are labeled). (b) The relative species composition for each dominant genotype. (c) River connectivity diagram showing one point per HUC8‐watershed connected to next downstream watershed. The blue portion of the diagram denotes the Columbia River (with lower and upper sub‐regions delimited by the confluence with the Snake River) and the green denotes the Snake River sub‐region (coastal sites are not included here due to general absence of connectivity with other watersheds). (d) The top left panel shows the connectivity matrix rotated at several nodes to form a balanced dendrogram that is used in the other panels where size of the red dots denotes the number of years in the 2000–2012 period in which that dominant genotype was detected in that HUC8 watershed (larger indicating more years)

A total of 90 genotypes were reported during the study, with 70 in adult fish and 35 in juvenile fish. Of these genotypes, 14 were found in both adult and juvenile fish, half of which were dominant types (see below). Among typed steelhead isolates, M group viruses were detected 1.8 times more frequently than U group viruses (176 M types, 96 U types), and among Chinook, U group viruses were detected 4.1 times more frequently than M group (39 M types, 158 U types). Consistent with previous reports, genotypes detected here did not occur at similar frequencies (Breyta et al., 2013; Breyta, Black, et al., 2016; Garver et al., 2003). The majority of genotypes (69 out of 90) were detected only at one site in a single year, while seven genotypes made up 77% of the genotyped records. These seven were detected in ten or more sites and in five or more years during our study and hereafter are referred to as dominant genotypes (Figure 5a). Two of these dominant genotypes are from the M group and were primarily detected in steelhead trout, while three dominant U group genotypes were primarily detected in Chinook (Figure 5b). Dominant genotypes mG032U and mG001U were detected in both Chinook and steelhead. Dominant genotypes demonstrated variable degrees of spatial heterogeneity during our focal study period (Figure 5d). Although some genotypes were widely dispersed across all three major sub‐regions shown in Figure 4c (i.e., mG001U, mG139M, and mG151U), others were more restricted to a single sub‐region (mG032U, mG110M, and mG174U). Likewise, the temporal frequency of detection varied across dominant genotypes during this study period (Figure 5d). Viral genotypes mG001U and mG110M were detected in 10 and 11 years, respectively, while mG147U was only seen in 6 years. This implies a range of temporal‐ or frequency‐based success, in that the types detected in more years appeared more successful.

### 3.5 Support for transmission scenarios

We used the IHNV‐VGS database to test for evidence in support of three possible transmission routes that we hypothesized may contribute to infection in juvenile fish cohorts. We first considered the total of 191 virus‐positive juvenile cohorts in the database and identified the subset for which there was a positive source cohort that was consistent with each transmission route. These subsets of candidate cases were then examined using available genotype data to identify which cases shared an identical genotype with the candidate source population, to indicate an upper bound on the estimate of how often this transmission route may have occurred. Within this subset, the proportion of most strongly supported cases was then identified where the identical genotypes were more informative because they were either rare genotypes or dominant genotypes detected outside their endemic spatial and temporal range (Figure 5d). For all transmission routes, genotype analyses also quantified evidence against a specific transmission route by defining the proportion of cases where genotypes of candidate source populations were different from those of the positive juvenile cohorts of interest.

### 3.6 Within‐hatchery transmission between juvenile cohorts (route 1)

One of the ways that IHNV may persist in the landscape is through inter‐cohort transmission between juvenile fish within a hatchery. Here, we simply ask whether or not within‐hatchery viral maintenance is likely. There were a total of 135 positive juvenile cohorts (71% of 191 total positive juvenile cohorts) that fit the criteria for route 1 by occurring as one of two or more consecutive year positive juvenile infections at the same hatchery site. Viral genotypes were available from 98 of these, revealing that 54 (55% of genotyped candidate route 1 cohorts) had identical genotypes (Table 2). Many of the genotypes detected in the consecutive juvenile cohort cases were dominant types that were also detected at other hatchery sites in the same time frame, thus providing only weak inference for transmission route 1. However, in 18 cohorts, detections of dominant genotypes provided moderate support due to their unusual patterns of occurrence (see Table 2 footnotes). There were also cohorts where detection of rare genotypes mG178M and mG206M provided strong evidence for transmission linkages, as they were only detected in the specific sites where the consecutive juvenile cohorts occurred (Table 2). However, these genotypes also occurred in adult fish at these sites and thus could indicate either pathway 1 or 3 (as detailed below). The informative genotypes collectively provided strong support that a minimum of 10 cases (10% of genotyped route 1 specific candidate positive juvenile cohorts) were likely to have been infected via route 1. The lack of matching genotypes in 44 cases (45% of genotyped candidate route 1 cohorts) of the consecutive infections indicated that transmission pathway(s) other than route 1 also likely contributed to infection in this subset of positive juvenile cohorts (Table 3).

**Table 2:** Cases of identical genotypes between consecutive year virus‐positive juvenile fish cohorts at the same hatchery and levels of inference provided by the genotyping to inform transmission scenario 1

Site	Host type(s)	Years (no. of juvenile cohorts)^a^	mG ### genotype	Transmission inference^b^
1	Sthd	2008, 2009	110M	Strong‐b
2	Sthd	2007, 2008	110M	Strong‐b
3	sthd, chin, rb	2006(3), 2007	110M	Strong‐a
4	sthd, chin, rb	2005, 2006(2), 2007	110M	Strong‐a
5	Sthd	2003, 2004	110M	Weak
6	Sthd	2003, 2004, 2009–2011	110M	Weak
7	Sthd	2008–2010	110M	Weak
7	Sthd	2004–2005	139M	Highest
7	Sthd	2009–2011	178M	Highest^1 or 3^
8	Chin	2001–2002	001U	Weak
9	sthd, chin, rb	2007, 2008	032U	Strong‐b
9	sthd, chin, rb	2002, 2003, 2005(2), 2006–2008	110M	Weak
10	sthd, chin	2004–2006	001U	Weak
11	sthd, chin	2003(2), 2004(2), 2005	001U	Weak
12	chin	2010, 2011	174U	Highest
13	sthd, chin, rb	2008, 2009, 2010(2), 2011	139U	Strong‐b
13	sthd, chin	2009–2010	206M	High^1 or 3^

sthd, steelhead; Chin, Chinook; rb trout, rainbow trout.

a Where no number is given in parentheses there was one juvenile fish cohort in that year.

b Weak inference occurs because the genotype detected was a dominant genotype found also in other possible sources of transmission; strong‐a means genotype was new to the HUC8 watershed; strong‐b means genotype was new to the wider sub‐region (Figure 5); highest means genotypes were not previously detected in any other location; highest ^1 or 3^ indicates cases where rare genotypes were found in both previous juvenile fish and returning adult fish, thus strongly supporting transmission but not distinguishing between routes 1 and 3.

**Table 3:** Summary of route‐specific inference

Transmission route	No. of candidate positive juvenile populations^a^	No. of candidate populations genotyped	No. of identical genotypes (% of number of cohorts genotyped)	No. of informative, supporting genotypes (% of number of cohorts genotyped)	No. of contradicting genotypes (% of number of cohorts genotyped)
Route 1	135	98	54 (55%)	10 (10%)	44 (45%)
Route 2	48	45	35 (78%)	14 (31%)	10 (22%)
Route 3	107	85	63 (74%)	22 (26%)	22 (26%)

There were a total of 191 positive juvenile cohorts during the study, and the subsets of these that fit the criteria for each transmission route are listed, along with how many of each subset were genotyped. Transmission routes are not mutually exclusive. Tallies of juvenile cohorts with candidate source populations that had identical genotypes, strongly supportive genotypes, or contradictive genotypes are shown, including the percent of number of cohorts genotyped.

a See Results for criteria used for each route.

As a separate analysis, we also considered possible transmission between concurrent juvenile fish cohorts in the same year within the same hatchery although in these cases the direction of transmission is not known. At 18 hatchery sites where more than one virus‐positive juvenile cohort was detected during the same year, the genotyped cohorts (12 out of a total of 31 positive) were examined for evidence of within‐hatchery transmission. In nine of these cases, juvenile fish from two or three different host types were IHNV positive with the same genotype, implying the possibility of some direct or indirect transmission within the hatchery setting. All types detected in concurrent infections were dominant genotypes (Table 3).

We also evaluated how hatchery program size, as proxied by numbers of juveniles released and numbers of species reared, influences IHNV recurrence (route 1 transmission). We hypothesized that larger hatcheries would be more susceptible to juvenile infection and thus, more likely to also have recurrent infections across cohorts. Hatcheries that reared more fish did report more years with positive IHNV samples of any life stage (glm, *z* = 4.63, *p* < .001) although this was not significant when we considered only positive juvenile records (glm, *z* = −0.26, *p* = .80). Likewise, there was no significant relationship between number of positive juvenile years and number of species reared at a given hatchery (glm, *z* = 0.64, *p* = .52).

### 3.7 Inter‐hatchery juvenile transmission (route 2)

If juvenile fish within a hatchery become infected from proximal hatcheries via contaminated effluent, shared biosecurity issues across nearby sites, or out‐migrating infected juvenile fish, then we would expect spatial clustering of juvenile infections. We evaluated this at the HUC8‐watershed level to identify infection patterns across nearby sites within a watershed. Watersheds (HUC8) with more hatcheries reported greater annual frequency of IHNV infections in juvenile fish (Spearman's rank correlation: rho = .449, *p* < 10^−5^). There were also frequent, concurrent IHNV infections in juvenile fish cohorts located in the same watershed, occurring in 91 (55% of 166 total) of instances where multiple hatcheries in a watershed tested juvenile fish.

As a clear indicator of possible route 2 transmission, distinct from route 1, we examined whether newly infected hatchery cohorts (i.e., those that had not reported IHNV‐positive juvenile samples within the previous year) were located in watersheds with concurrent juvenile infection at nearby sites (upstream or downstream) in the same year or prior year. The strongest evidence for route 2 was observed in 20 (22%) out of 91 newly infected site‐cohorts, where juveniles at another hatchery in the same HUC8 watershed were positive in both the concurrent and previous year. Another 28 (31%) of the new infections occurred all at the same time, that is, without any juvenile infections in the HUC during the previous year, which is consistent with either route 2 or route 3 transmission. The remaining 43 (47%) of new juvenile infections were isolated events that occurred in the absence of any other juvenile infections within the watershed in either the concurrent or previous year, implying the involvement of other transmission routes.

Of the 48 newly infected site‐cohorts consistent with route 2 inter‐hatchery transmission, 45 (94%) were genotyped, and identical genotypes were found in 35 cases (78% of genotyped candidate route 2 cohorts). Among the 20 cases where candidate nearby juvenile sources occurred in the previous and concurrent year, 19 cohorts were genotyped and 10 of these provided additional support for this transmission route. These consisted of eight cohorts with dominant genotypes, including three cases where these provided strong support due to unusual occurrence, and two cases of rare genotype detection that were strongly supportive. The rare genotype mG157M emerged in juvenile fish and was then found in juvenile fish at another hatchery within the same HUC8 watershed, whereas genotype mG168M emerged in juvenile fish and was then detected in nearby adults, possibly indicating juvenile‐to‐adult transmission between proximal hatcheries. Among the other 28 cases where candidate sources occurred only within the concurrent year, 22 cohorts were genotyped but only 4 supported transmission via route 2. Informative genotype evidence therefore supports 14 cases (31%) of route 2 transmission. Interestingly, the genotype data also provided evidence that a transmission route other than route 2 was acting in another 10 cases because genotypes of candidate source populations did not match the new infection, indicating that 22% of new juvenile infections are not likely explained by route 2 transmission (Table 3).

### 3.8 Transmission from returning adults (route 3)

Adult fish returning to spawn are frequently reported as IHNV positive and these fish are widely considered to be a likely source of virus transmission to juvenile hatchery fish (Anderson et al., 2000; Bootland & Leong, 1999; Breyta, Samson, et al., 2016; Emmenegger et al., 2000). However, the relative importance of this pathway has not been previously quantified. We examined the IHNV‐VGS database for evidence of this transmission route at discrete hatchery sites and found that 109 of the 169 hatchery sites reported testing both adult and juvenile fish within the same year at some time during the study period. At these sites, there were 121 positive juvenile cohorts, and adult cohorts were positive in the same or previous year for 107 (88%) of these (adults tested at upstream sites were not analyzed). Genotype data revealed identical genotypes in 63 of these cases (74% of genotyped candidate route 3 cohorts). Strong support for route 3 transmission was found in 22 (26%) of the cases: 16 cases where dominant genotypes were found in informative circumstances and in 6 cases of rare genotypes detection. These three non‐dominant genotypes were mG157M, mG178M, and mG206M all of which were limited to the locations where they were first detected. There were 22 (26%) cases where genotyping data suggested that a different transmission route was acting (Table 3).

## 4 DISCUSSION

Work presented here represents the first landscape‐scale epidemiologic analysis of IHNV surveillance data in the Columbia River Basin and adjacent coastal rivers. Our summary analyses confirm that both juvenile and adult salmonids are likely involved in maintaining a persistent presence of IHNV in Pacific Northwest ecosystems. This is an important conclusion, as it suggests that management of the virus must address both the propensity for within‐hatchery transmission between juvenile cohorts and the rather more difficult issue of infectious adult fish. Managing within‐hatchery transmission can be effectively controlled using strict biosecurity measures. However, the risk posed by adult fish is largely as an infectious virus‐shedding contaminant of hatchery water supply, since egg disinfection is widely used to block parent‐to‐offspring transmission. The presence or absence of susceptible species in a culture facility's water supply is generally referred to as water supply security (an unsecure water supply contains susceptible species), and many hatcheries were built before the importance of water supply security was recognized. Changing such fundamental infrastructure as the water supply is extremely difficult, though not unprecedented (Breyta, Samson, et al., 2016). Once disease occurs, the management strategies imposed are so variable and influenced by so many factors that they are beyond the scope of this report.

Over the 13 years examined here, IHNV was detected at relatively stable prevalence levels that ranged from 8% to 30% of all tested fish cohorts, in various age and host type sectors of its Pacific salmonid multi‐host complex. This is a relatively high landscape prevalence for a viral pathogen, reminiscent of the 10%–27% prevalence range reported for human immunodeficiency virus (HIV) in southern African countries that have the highest burden of HIV in the world (Global report 2012). While the IHNV data presented here are prevalence among all cohorts instead of within a cohort, the fact remains that IHNV is present at levels well above “rare.” These IHNV prevalence levels, and the high mortality often associated with infection of juvenile fish, confirm the role of IHNV as a major pathogen of salmonid fish that continues to influence the success of conservation programs within the Pacific Northwest. The highest overall prevalence (in both adult and juvenile fish) of IHNV infection in the study region occurred in steelhead trout. Twenty‐nine percent of the hatcheries rearing steelhead trout during the study period reported at least one positive cohort year. When it does emerge in a hatchery, IHN disease has caused high mortalities in steelhead (Bootland & Leong, 1999; Breyta et al., 2013; Breyta, Samson, et al., 2016).

One of the most outstanding questions in the management of this viral pathogen is the mechanism(s) of emergence. Here, we define emergence as the appearance of recognized viral strains in a new host type, like steelhead (Groberg et al., 1982), or in a new geographic region (Breyta et al., 2013), or the emergence of new viral strains that have increased virulence (Breyta, McKenney, et al., 2016). This definition assumes that homoplasy at the level of viral gene sequence is not occurring. There have been several well‐documented periods of emergence of IHNV, most of which were associated with significant mortality (Breyta et al., 2013; Breyta, Black, et al., 2016; Garver et al., 2003; Kurath et al., 2003; Troyer et al., 2000). On a landscape scale, then, a critical and poorly understood factor is the primary mode of transmission to juvenile fish. Since many populations of salmonids in the Pacific Northwest are semi‐cultured, existing management practices could be adapted to better disrupt the primary transmission route if it were known. Therefore, we synthesized information from the IHNV surveillance database in support of testing three proposed transmission pathways that could be responsible for infections in juvenile hatchery fish. At this time, we have not addressed transmission to adult fish, or to wild fish, but instead focused on juvenile hatchery fish, where the majority of observed IHN disease events occur. We demonstrate that within‐hatchery viral maintenance by transmission between consecutive juvenile fish cohorts (route 1) is estimated to explain a minimum of 10%, and at most 55% of the candidate positive juvenile cohorts where this route was possible (Table 3). Some of these sites of recurrent juvenile infections are larger hatcheries (Breyta, Black, et al., 2016), rearing fish at high densities, or more than one species of juvenile fish; however, there was no consistent relationship between hatchery size and the probability of juvenile infection. Inter‐hatchery transmission between juvenile fish (route 2) was found to explain a minimum of 31%, and at most 78% of candidate cases. The transmission of virus from returning adult fish to hatchery juvenile cohorts (route 3) was estimated to explain a minimum of 26%, and a maximum of 74% of the candidate cases where this route was possible. Collectively, these results suggest that each of the routes tested functions within the study region and accounts for a non‐trivial proportion of virus transmission, and none of them alone account for all possible cases of transmission to juvenile fish cohorts (Table 3). The results suggest that both infected juvenile fish and infected migrating adult fish are likely to play important roles in moving the virus across the landscape and between hatcheries. The observation that infections in returning adult populations may be one mechanism serving to maintain focal spots of juvenile infection is consistent with previously published case studies that found matching IHNV genotypes across life history stages at a specific site, and suggested that infected adults in the water supply of a hatchery are direct sources of viral transmission to juvenile cohorts (Anderson et al., 2000; Bendorf et al., 2007; Breyta, Samson, et al., 2016; Emmenegger et al., 2000). To our knowledge, specific data with viral genotype support that demonstrates probable transmission between juvenile fish has not been previously reported.

Our analysis also indicated lower IHNV prevalence in wild fish relative to the same species of fish reared in hatcheries although this observation has several caveats. Even with the large database at hand, we are unable to conclude whether this lower prevalence reflects lower transmission rates to wild fish populations or a sampling bias. It is possible that wild fish are not exposed to similar levels of virus as hatchery fish due to differences in environment. However, the lower prevalence in wild fish could also be due to one or more confounding factors. First, the wild fish surveillance program is more opportunistic in terms of fish numbers, and samples fish species in different proportions (including those known to have a low burden of IHNV (e.g., coho salmon). Second, the sampling of wild fish is inherently biased toward healthy fish because unhealthy or dead fish are not as likely to last long enough in the environment to be sampled. Thus, wild fish sampling usually detects evidence of infection rather than disease. Since predators may target moribund wild fish, prevalence of IHN disease may be underestimated. Also, wild fish populations are generally under‐surveilled and transmission of IHNV between hatchery and wild fish populations is difficult to assess (Kurath & Winton, 2011). Limited data on wild fish populations impede our ability to determine whether infections in wild fish are infrequent spillover events or whether wild fish serve to maintain IHNV across the region. Furthermore, no genotype data were available for virus‐positive records from the NWFHS wild fish testing. Thus, enhanced surveillance of wild fish and genotyping of wild fish virus isolates could provide important inferential power for linking wild fish infections with nearby hatchery‐based virologic and genetic surveillance and should be a priority in future efforts.

Genotyping results were available for the majority of positive cohorts from hatchery sites, allowing added inference of possible transmission scenarios (Table 3). In the logic used for interpreting genotype data, both matching and non‐matching genotypes are informative. For a specific transmission route, support is achieved when genotypes are identical, and the strength of the support varies depending on whether the genotypes are dominant or rare. If the genotypes do not match, then there is no support for the scenario and we can conclude that some other route of transmission was likely responsible for the juvenile infection in question. On a landscape scale, the proportion of relevant cases that have matching or non‐matching genotypes provides a quantitative estimate of how often the transmission may have occurred or likely did not occur, respectively. Due to the widespread nature of some genotypes, however, there is a gradient of inferential power in matching genotype cases, where common genotypes outside endemic range and cases involving rare genotypes are the most informative. For each transmission route tested, 22%–45% of positive cohorts showed evidence of some other transmission route acting. This does not imply transmission routes other than the three tested here, it may simply mean that candidate source populations other than those sampled here were involved in transmission. For example, a caveat for the route 2 analysis presented here is that we considered only new infections, in order to preclude the simultaneous possibility of route 1 transmission. However, route 2 may also be responsible for infections that are not new in a hatchery, as we observed cases where new genotypes appeared and then spread among sites within a HUC, apparently replacing previously detected genotypes. Furthermore, route 3 transmission may have contributed to any candidate cases of route 2 analyzed here. An important caveat for the route 3 genotype analysis is that our methods did not consider the possibility of viral exposure from waterborne virus shed by adults returning to upstream hatchery sites although adult fish are known to hold stationary at times during their return migrations depending on environmental conditions. A general caveat relevant to the independent analyses of the three transmission routes tested here is that the routes are not mutually exclusive, and in many cases even strong genotype support cannot conclusively support only one possible route, as in the detection of rare genotypes mG178M and mG206M (at sites 7 and 13 in Table 2), where either route 1 or 3 was shown to be possible. This reflects the complex ecology of the Pacific salmon multi‐host assemblage for IHNV, where juvenile and adult life stages occur regularly in sufficient proximity to facilitate virus transmission.

There are potential issues with interpreting genotype data that we must consider in evaluating transmission routes at this landscape scale. Dominant genotypes provide weaker evidence for a transmission route than do rare genotypes. Out of 143 genotyped positive juvenile cohorts in this study, there were only six cases where a rare genotype (i.e., not one of the 7 dominant strains in Figure 5) provided strong support for a particular transmission route. Specifically, these included evidence of adult to juvenile transmission of genotypes mG139M and mG174U and a case where juveniles at one site likely transmitted type mG157M to juveniles at a downstream site. Additional examples of likely juvenile‐to‐adult transmission occurred in cases with genotypes mG168M, mG178M, and mG206M. A greater capacity for including genetic variation in transmission inference, like a weighted genetic similarity instead of strict genetic identity, may be necessary to maximize the value of the genotyping at this landscape scale.

The transmission pathways analyzed here were designed to provide inference regarding how juvenile fish become infected with IHNV, but the involvement of adults in the introduction and persistence of IHNV also raises several questions about how adult fish become infected. The capacity for infected adults to shed infectious virus is not well documented, and it may vary by host species or even between viral genotypes. Semi‐cultured adult fish are targeted for IHNV screening throughout the study region, regardless of the historic presence of virus in a given fish population's history, but this sampling is lethal and therefore only conducted at the end of the freshwater migration. Susceptibility to IHNV infection may increase during spawning, when the immune function is known to wane (Schreck, 1996). Alternatively, the depressed immune function may allow chronic/latent IHNV from an early‐life infection to resume active replication, as suggested in the paper by Bootland and Leong (1999). Transmission among ocean‐dwelling immature adult fish is also possible, but has never been documented. Regardless of the infection pathway, adult fish seem to be a likely critical link in the transmission cycle that maintains IHNV prevalence in the study area. This suggests that they may provide a point in the cycle that may be targeted for control measures. Indeed, two case studies have shown that if juvenile fish are protected from water that harbors infected adults, the cycle of transmission was disrupted and juveniles did not suffer epidemic IHN disease, for as long as the control strategy was studied (Bendorf et al., 2007; Breyta, Samson, et al., 2016).

The IHNV pathogen–host system has a complex disease ecology that involves multiple host species, each with differing susceptibilities and genetically distinct subpopulations, and a rapidly evolving virus covering a wide range of virulence profiles (Breyta et al., 2014; Breyta, McKenney, et al., 2016; Brieuc, Purcell, Palmer, & Naish, 2015; Garver, Conway, & Kurath, 2006; LaPatra, Fryer, & Rohovec, 1993; Peñaranda et al., 2009, 2011; Purcell et al., 2009). Wild‐ and semi‐cultured hatchery populations are often conspecific within species, and sympatric among species, but their pathogen transmission routes are largely uncharacterized. Laboratory experiments coupled with models that test the impact of different transmission mechanisms are needed to better understand the relative contributions of juvenile and adult fish in landscape spread of IHNV. Additional factors that likely contribute to IHNV disease ecology include differences in susceptibility of specific salmonid populations, cross‐species transmission, and additional anthropogenic mechanisms of transmission, including infections mediated by exchange of fish between hatcheries, artificial transport of juvenile and adult fish around barriers such as dams, or by the release of animals into different watersheds.

The data and results presented highlight common challenges in documenting and understanding epidemiology in semi‐cultured animals. Population size, dispersal, and infection status are all sampled and estimated with error. Thus, care is required when analyzing and interpreting results. At the same time, the need to manage or intervene in disease outbreaks can be urgent and thus, analysis and interpretation must be performed despite these caveats (Wasserberg, Osnas, Rolley, & Samuel, 2009). The dataset presented here is comprehensive in its scope, including all regional agencies operating within the study area, and it is unique in analyzing both positive/negative and genetic surveillance records of an aquatic pathogen. While much of the results are unique to the IHNV system at this point, there are numerous viral, bacterial, and cellular pathogens of Pacific salmon or other fish in aquatic environments that may share some or all of the transmission features reported here. The landscape scope of transmission and evaluation of testing efforts are critical components across all animal‐pathogen systems. In comparison with previous phylogeographic studies of IHNV in the Columbia River Basin that were based solely on virus genotyping data (Breyta, Black, et al., 2016; Garver et al., 2003), we show here that incorporating a landscape of negative surveillance results has important implications for how data on virus presence, dispersal, and genetic diversity are interpreted. The IHNV system is also a useful model for understanding how human management practices can interact with natural animal life history to influence the impact and persistence of disease in a regional landscape. This level of understanding is necessary for identifying effective interventions and monitoring their success.

## AUTHOR'S CONTRIBUTIONS

RB collected the data, IB georeferenced the data, RB, IB, and SL analyzed the data; PF, AW, GK, MP, and KN conceived ideas and designed methodology; RB, GK, IB, and SL led the writing of the manuscript. All authors contributed critically to the drafts and gave final approval for publication.

## CONFLICT OF INTEREST

No authors declare any conflict of interest.

## DATA ACCESSIBILITY

The data used in this study are available at https://doi.org/onlinelibrary.wiley.com/doi/10.1002/ecy.1634/full, https://doi.org/10.1002/ecy.1634
